# Avoiding risk at what cost? Putting use of medicines for breastfeeding women into perspective

**DOI:** 10.1186/1746-4358-7-14

**Published:** 2012-10-17

**Authors:** Lisa H Amir, Kath M Ryan, Susan E Jordan

**Affiliations:** 1 Mother & Child Health Research, La Trobe University, Melbourne, VIC, Australia; 2 School of Nursing and Midwifery, La Trobe University, Bundoora, VIC, Australia; 3 Department of Nursing, College of Human and Health Sciences, Swansea University, Swansea, Wales, UK

**Keywords:** Breastfeeding, Lactation, Medication, Side-effect, Risk, Maternal health, Postpartum

## Abstract

Breastfeeding women often need to take medicines, and therefore health professionals need to consider the effects of medication on lactation and the breastfed infant, and any associated risks. This commentary discusses the tragic case of a young woman with a history of mental illness who committed suicide in the postpartum period. She was determined to be a 'good mother' and breastfeed, and to avoid any potential adverse effects of medication on her breastfed infant. The final outcome was fatal for both mother and child. We argue that if women require medication during lactation, all risks need to be considered – the risk of not treating the maternal medical condition may greatly outweigh the potential risk to the breastfed infant.

## Commentary

The death of the brilliant and empathetic young UK psychiatrist who committed suicide in 2000 can have a positive impact on maternal and child health if we learn from the family tragedy [1]. An editorial in *The Psychiatrist* describing the obstacles experienced by medical professionals seeking and receiving help for mental illness has highlighted this case again [2].

The North East London NHS report on the extended suicide of Dr Daksha Emson, and her three month old daughter, Freya, describes her history of bipolar affective disorder and itemises the potential factors that contributed to the disastrous outcome: immigration, racism, childhood sexual abuse, stigma of mental illness, being a doctor and a patient, and the stresses of pregnancy losses and the postpartum period [3]. She had overcome many obstacles in order to establish her medical career; at times, alternating between hospital admissions and academic prizes.

Dr Emson had stopped her medications when planning a pregnancy, and was reluctant to restart postpartum as she was keen to breastfeed her daughter.

"“Being a *good mother* by breastfeeding Freya, as Daksha saw it, took such precedence over the need for treatment of her mental illness that she put her mental health at risk. There is a great deal of ignorance about breastfeeding and bipolar affective disorder on the part of professionals and conflicting views by expert witnesses about whether it is safe to breastfeed in conjunction with a mood stabiliser such as lithium carbonate.” [3] p. 19] (our emphasis)."

We shall never know if this double tragedy could have been averted if Dr Emson had been more closely monitored in the risky postpartum period, and if she had recommenced lithium treatment. In the past, lithium was regarded as one of the few medicines contraindicated for breastfeeding women [4]. It was thought the risk to the breastfed infant was too high.

Today, however, clinicians are looking more closely at the compatibility of medicines such as lithium and breastfeeding. Recent guidelines from *beyondblue* in Australia state that “If the woman chooses to breastfeed, lithium should be used with particular caution. The decision should be made in consultation with a specialist physician and, where possible, there should be ongoing specialist monitoring for potential adverse effects on the breastfed infant." [5] p. 56]. Case series have shown that infants’ lithium concentrations are not as high as previously thought, and if infants’ plasma concentrations are monitored, continued breastfeeding is possible in selected women [6,7]. Debra Bogen and colleagues have reported three cases of women who successfully breastfed while maintaining lithium treatment [8]. If a woman wishes to breastfeed during lithium therapy, the neonate must be closely observed for dehydration, tremor, involuntary movements, cyanosis, muscle tone and other adverse effects, and monitored by blood tests for lithium concentrations [6,7]. Supplementary formula feeds should be available in case toxicity is suspected.

The ‘good mother’ discourse asserts that good mothers will breastfeed and avoid any possible contaminants entering their bodies or their breast milk [9]. Health professionals need to challenge this assertion. Good mothers need to look after their own health first, and not compromise their treatment options. Evidence shows that women with mental illnesses are not able to function optimally [5], yet are cautious about taking medicines while breastfeeding because of fear about the effects on their infant or on their ability to breastfeed [10,11].

The majority of breastfeeding women do take medication [12,13], as women face both acute illnesses and ongoing medical conditions in the postpartum period. However, the impact on breastfeeding rates, breastfeeding success and infants’ outcomes is largely unknown [14-18].

To advise women about the compatibility of medicines and breastfeeding, health professionals need access to accurate information [19]. Information from pharmaceutical companies is usually unnecessarily cautious and should not be relied on. Although more research is urgently needed, clinicians can access information electronically through LactMed [20] and the Perinatal Psychotropic Medicine Information Service [21], as well from information services and books [22-24]. Larger studies and long-term follow-up data on child health and school performance, which we are currently lacking, are needed to improve advice for parents [17,25-28]. Another factor limiting clinicians' decision-making about medicines in lactation is that it is almost impossible to access measurement of drug levels in maternal milk or infant blood. Real time access to measurement of drug levels in clinical laboratories would facilitate evidence-based decisions.

Women and their families need to be aware that depression and anxiety are common in the postpartum period. Women may not realise that feelings of exhaustion, sadness, anxiety, loss of appetite and difficulty sleeping may be symptoms of depression and related conditions [5]. Health professionals should discuss these issues with new mothers and offer information, support and treatment options.

Most medicines are safe for breastfeeding women as the breastfed infant would receive less than 3% of a therapeutic dose per kilogram of body weight [25] p. 617], and many medicines are prescribed for neonates themselves. When the decision is not straightforward, clinicians need to consult experienced pharmacists before concluding that breastfeeding and medicine use is too risky. Most decisions won’t be as difficult as prescribing lithium in the peripartum period, but Dr Daksha Emson’s tragic case should teach us that failing to prescribe medicine for a breastfeeding woman could be fatal for both mother and child.

## Competing interests

The authors declare they have no competing interests.

## Authors' contributions

LHA wrote the first draft of the paper. The other authors revised the paper with additional intellectual input. All authors approved the final version.
